# The relationship between training status, blood pressure and uric acid in adults and elderly

**DOI:** 10.1186/1471-2261-13-44

**Published:** 2013-06-21

**Authors:** Atila Alexandre Trapé, André Mourão Jacomini, Jaqueline Jóice Muniz, Jonas Tadeu Cau Sertorio, José Eduardo Tanus-Santos, Sandra Lia do Amaral, Anderson Saranz Zago

**Affiliations:** 1 Faculty of Medicine, University of São Paulo, Ribeirão Preto, SP, Brazil; 2 Department of Physical Education Unesp, São Paulo State University, Bauru, SP, Brazil

**Keywords:** Blood pressure, Uric acid, Training status, Elderly, Oxidative stress, Nitric oxide

## Abstract

**Background:**

Hypertension can be generated by a great number of mechanisms including elevated uric acid (UA) that contribute to the anion superoxide production. However, physical exercise is recommended to prevent and/or control high blood pressure (BP). The purpose of this study was to investigate the relationship between BP and UA and whether this relationship may be mediated by the functional fitness index.

**Methods:**

All participants (n = 123) performed the following tests: indirect maximal oxygen uptake (VO_2_max), AAHPERD Functional Fitness Battery Test to determine the general fitness functional index (GFFI), systolic and diastolic blood pressure (SBP and DBP), body mass index (BMI) and blood sample collection to evaluate the total-cholesterol (CHOL), LDL-cholesterol (LDL-c), HDL-cholesterol (HDL-c), triglycerides (TG), uric acid (UA), nitrite (NO2) and thiobarbituric acid reactive substances (T-BARS). After the physical, hemodynamic and metabolic evaluations, all participants were allocated into three groups according to their GFFI: G1 (regular), G2 (good) and G3 (very good).

**Results:**

Baseline blood pressure was higher in G1 when compared to G3 (+12% and +11%, for SBP and DBP, respectively, p<0.05) and the subjects who had higher values of BP also presented higher values of UA. Although UA was not different among GFFI groups, it presented a significant correlation with GFFI and VO_2_max. Also, nitrite concentration was elevated in G3 compared to G1 (140±29 μM *vs* 111± 29 μM, for G3 and G1, respectively, p<0.0001). As far as the lipid profile, participants in G3 presented better values of CHOL and TG when compared to those in G1.

**Conclusions:**

Taking together the findings that subjects with higher BP had elevated values of UA and lower values of nitrite, it can be suggested that the relationship between blood pressure and the oxidative stress produced by acid uric may be mediated by training status.

## Background

The growing number of the elderly population and their complex health problems reveal the necessity of creating adequate health care programs. Among the elderly, the prevalence of hypertension reaches about 50% and has been considered the main risk factor for cardiovascular diseases. Therefore, the comprehension of the different mechanisms that generate hypertension is fundamental for creating strategies to control high blood pressure (BP).

It has been shown that elevated uric acid (UA) can be one of the mechanisms responsible for hypertension. It is the final enzymatic and product of purine degradation in the human body and it is catalyzed by xanthine oxidase [1]. Some authors have shown that low mitochondrial capacity can increase xanthine oxidase activity which will further induce UA formation, thus favouring anion superoxide (O_2_^-.^) production [2] which can lead to endothelial dysfunction and vascular damage [3,4]. Elevated levels of UA can be associated with NO-scavenging, lower vasodilator response [5] and higher BP levels [6]. Accordingly, Zharikov et al. [7] showed that almost 80% of patients with pulmonary hypertension had hyperuricemia. However, regular physical exercise has been considered the major stimulus to prevent and/or control high BP [8,9]. Briefly, physical exercise promotes an improvement in metabolic syndrome [10], autonomic modulation (heart-rate variability) [11], nitric oxide concentration and oxidative stress [4,9], lipid profile [12] and other disorders. Nevertheless, the effectiveness of regular exercise on UA content and its relationship with hypertension is still controversial, mainly because of the differences among type, intensity, frequency and duration of the exercise programs found in the literature [13-15], especially those related to elderly [5,14,16].

In addition, the current recommendation for elderly people has been recognized as a combination of different types of exercise for maintaining physical functional fitness [14]. Both the American College of Sports Medicine [14] and the American Heart Association (AHA) [15,16] recommend that the exercise prescription for adults and elderly people should include endurance, muscle strengthening as well as balance and flexibility exercise (also called multicomponent exercises).

Following these recommendations, some studies compared the effects of a multicomponent exercise program with a strength exercise program [12] and with a walking program [13]. These studies suggested that better results on lipid profile can be found after a multicomponent exercise program compared to others. Therefore, it seems reasonable to suggest that results from a multicomponent functional fitness test are more appropriate to define training status instead of the evaluation of only one component of physical fitness, like cardiovascular capacity [12,17].

Although it has been suggested that people with sedentary lifestyle exhibit lower mitochondrial capacity [18], there is limited evidence about the relationship among training status, BP and UA. Thus, the hypothesis of this study was that people with better training status could have lower BP and UA levels. Therefore, the purpose of this study was to investigate the relationship between BP and UA and whether this relationship may be mediated by the training status.

## Methods

### Screening

All the procedures were previously approved by Institutional Review Board of University of São Paulo / Brazil (CEP/FCFRP n°.172) and all the subjects provided written consent before the beginning of experiments.

The sampling plan was probabilistic and obtained in two steps: the first one was obtained by random selection of clusters (associations of retirees and programs linked to universities and City Hall of Ribeirão Preto / SP) that constitute and reflect the heterogeneous characteristics of the whole population. In the second, all individuals of each cluster were invited to participate in this study, with the same chance to be included once they met the following inclusion criteria: subjects should be non-smoking, non-alcoholic (<3 drinks/beers per day), age between 55–70 years old, non-diabetic (fasting glucose level < 100 mg/dL), should not have cardiovascular (angina, vascular disease, etc.), peripheral, cerebrovascular, neurologic or psychiatric diseases, should have a body mass index <35, should not be under treatment with medications known to affect glucose metabolism or renal hemodynamics, not have maximal systolic blood pressure (SBP) > 160 mmHg and maximal diastolic blood pressure (DBP) > 100 mmHg or other medical or orthopedic conditions which could affect their ability to successfully participate in a physical exercise battery test. The subject medical history and all assessments were reviewed on their first visit.

Blood samples were drawn after a 12-h overnight fasting for further analysis of lipid profile and uric acid content. All participants were informed to avoid fatty and hypercaloric food in the last meal before the 12-h fasting prior to blood collection.

All subjects underwent a physical examination to assess the body mass index (BMI) and blood pressure (BP). After that, training status was evaluated by both indirect maximal oxygen uptake test ($\dot{V}O_{2}\max$) and a “Battery Test” proposed by the “American Alliance for Health, Physical Education, Recreation and Dance” (AAHPERD) in order to assess the functional fitness.

Baseline Testing (performed at least 24h after the last exercise session):

a) Blood samples were used to evaluate the total-cholesterol (CHOL), low-density lipoprotein cholesterol (LDL-c), high-density lipoprotein cholesterol (HDL-c), triglycerides (TG) and uric acid (UA) by enzymatic-colorimetric methods (Laborlab commercial kits, Guarulhos, São Paulo, Brazil). Whole-blood nitrite concentration (NO2) was analyzed with ozone-based chemiluminescence (Sievers Model 280 NO Analyzer, Sievers, Boulder, CO, USA), as previously described [19]. Plasma lipid peroxide levels were determined by measuring thiobarbituric acid reactive substances (T-BARS) using a fluorimetric method as previously described [20]. The lipoperoxide levels were expressed in terms of malondialdehyde (nmol/mL).

b) Blood pressure (BP) was measured after 5 min of rest between 7–8 am on three separate days according to the VI Brazilian Hypertension Guidelines (SBH, 2010), using an aneroid sphygmomanometer adequate to the circumference of the arm and a stethoscope placed over the brachial artery. The average of the three separately recorded BP measurements was used as the outcome variable on data analysis.

c) Training status: All participants performed two tests in order to determine the training status:

(1.) The one-mile walk test as described previously [21]. Briefly, the test consisted of walking a 1 mile distance, without running in the shortest possible time. To calculate the VO_2_ max the following equation was used: VO_2_max = 6.9652 + (0.0091*weight) - (0.0257*age) + (0.5955*sex) - (0.2240*time) - (0.0115*final heart rate). The one-mile walk test protocol provides a valid sub-maximum assessment for VO_2_ max estimation (r=0.93). The option to use this test was because walking on a running track is closer to the daily activities as compared to walking on the treadmill;

(1.) The AAHPERD functional fitness test battery was used to assess the functional fitness of the participants. The following tests were included: Coordination – time to complete a task requiring a manipulation of 12-oz soda cans in a precise fashion; Flexibility – sit-and-reach test; Muscular strength and endurance reported as the number of biceps-curl repetition performed during 30 s (4lbs. for women, 8lbs. for men); Agility dynamic test – time to complete tasks involving repeatedly standing from a chair, walking around cones, and returning to the chair; and cardiovascular endurance represented the time to complete an 880-yard walk. The AAHPERD battery test was completely described previously [22,23]. The GFFI was calculated using the sum of the percentile score of each test as described previously [22]. All items included in the AAHPERD Battery Test have demonstrated good reliability and validity for use in this age group. The test-retest reliability coefficients for the item in this test battery have been reported in the range of r = 0.80-0.99 [23]. All participants were divided according to the GFFI results (G1 – regular GFFI: 200 to 299 points / G2 – good GFFI: 300 to 399 points / G3 – very good GFFI: 400 to 500 points). No participant was classified as very weak GFFI (0 to 99 points) or weak GFFI (100 to 199 points).

### Statistical analysis

The sample size was calculated taking into account that the prevalence of hypertension is around 30% in the Brazilian population [24]. This calculation is based on an 80% power and significance level of 5% as described previously [25,26].

Descriptive statistics was calculated and Pearson’s correlation coefficient was performed to detect correlation among variables. One-way ANOVA with Scheffe post-hoc test was performed to assess statistically significant differences among groups. GFFI was considered as an independent variable.

The general linear model was used in order to adjust for BMI and age in all variables, since they were considered as confounding factors. The data were analysed using SPSS 17.0 statistical package. All results are presented as means (SD). Differences were considered statistically significant if p<0.05.

## Results

**Figure 1:**
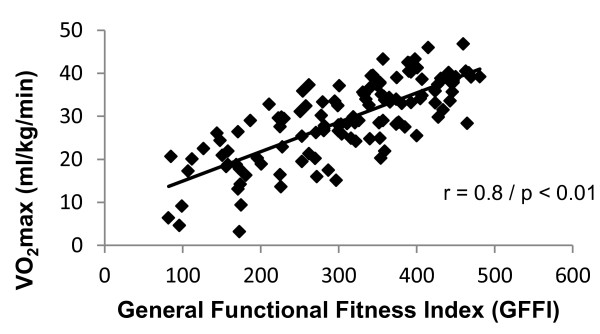
**Pearson correlation coefficient between general functional fitness index (GFFI) and**$\dot{V}O_{2}\text{max}$**.** *Correlation is significant at the 0.01 level.

From all the clusters visited, 123 participants met our initial inclusion criteria (17 men and 106 women). All participants were separated according to the GFFI index (G1 = 207.3 ± 62.7 / G2 = 351.2 ± 28.6 / G3 = 437.2 ± 23.2). The groups were composed of both genders even though the number of men was very low. The option to use the GFFI as an independent variable was because it comprises a multicomponent assessment, had a good correlation with VO_2_max (Figure 1), and also correlated with more variables than VO_2_max, as shown in Table 1. Therefore, this index seems to be more adequate to indicate the training status.

**Table 1:** Pearson correlation coefficient among variables

	**GFFI**	$\dot{V}O_{2}\max$
Age (years)	−0.38**	−0.61**
Total-cholesterol (mg/dL)	−0.35**	−0.25**
LDL-cholesterol (mg/dL)	−0.06	−0.06
HDL-cholesterol (mg/dL)	0.22	0.18*
Triglycerides (mg/dL)	−0.43**	−0.30**
Uric Acid (mg/dL)	−0.24**	−0.22*
Body Mass Index (kg/m^2^)	−0.22*	−0.28**
Systolic Blood Pressure (mmHg)	−0.43**	−0.37**
Diastolic Blood Pressure (mmHg)	−0.34**	−0.20*
Nitrite (μM)	0.31**	0.16
T-BARS (μM)	0.15	0.13

* p<0.05 and **p<0.01.

**Figure 2:**
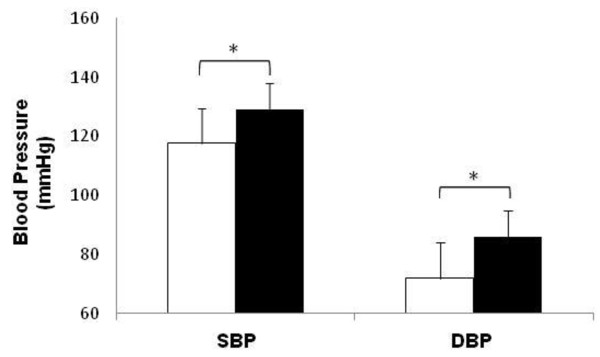
**Systolic (SBP) and diastolic (DBP) blood pressure in participants with normal uric acid (UA) values (white bars) and elevated UA values (black bars).** *p<0.05 (Student’s t-test). According to American Heart Association the normal values of UA should fall between 3.0 and 7.0 mg/dL. However, according to the commercial kit used in blood analyses, the normal values should fall between 2.5 and 5.0 mg/dL. Therefore, in the present study the UA values between 2.0 – 5.0 mg/dL were considered normal values and higher than 5.0 mg/dL were considered elevated values (p<0.05).

Pearson’s correlation coefficient also showed moderate correlation between UA and SBP (r=0.3/p<0.001) and DBP (r=0.29/p<0.001). Moreover, participants with elevated UA also exhibited high blood pressure values (Figure 2). Although BMI and age were considered as confounding factors, when participants were divided according to the UA levels the values of BMI (26.7 ± 4.0 and 28.5 ± 3.8) and age (56.7 ± 9.8 and 59.4 ± 11.6) were similar in both groups (low and high level of UA respectively).

In order to demonstrate how training status can mediate the relationship between blood pressure and uric acid, all participants were divided into three groups according the GFFI results. However, due to the difference among groups in BMI (G1 = 28.2 ± 4.2 / G2 = 27.4 ± 3.5 / G3 = 24.6 ± 3.5) and age (G1 = 60.9 ± 11.7 / G2 = 55.3 ± 8.1 / G3 = 53.9 ± 9.1) General Linear Model was performed to adjust for BMI and age. Subjects’ characteristics are presented in Table 2.

**Table 2:** Subjects’ characteristics

	**G1(n=51)**	**G2 (n=47)**	**G3 (n=25)**	**p**
Total-cholesterol (mg/dL)	203.4 (36)	183.0 (29)^a^	168.9 (31)^a^	0.000
LDL-cholesterol (mg/dL)	130.9 (32)	136.8 (21)	107.6 (16)	0.433
HDL-cholesterol (mg/dL)	43.6 (8)	47.5 (10)	47.5 (8)	0.072
Triglycerides (mg/dL)	157.7 (59)	110.2 (44)^a^	94.2 (39)^a^	0.000
$\dot{V}O_{2}\max$ (ml/kg/min)	22.1 (8)	32.8 (6)^a^	36.9 (5)^ab^	0.000
NO2 (μM)	111 (29)	119.8 (26)^a^	140.4 (29)^ab^	0.000
SBP (mmHg)	129.3 (14)	118.5 (15)^a^	114 (12)^a^	0.000
DBP (mmHg)	83.4 (14)	77.6 (9.6)^a^	74.2 (8.9)^a^	0.003
TBARS (μM)	2.25 (1.2)	2.33 (0.9)	2.49 (0.8)	0.640
UA (mg/dL)	4.8 (1.5)	4.2 (1)	3.8 (1)	0.104

GFFI, general functional fitness index; G1, regular GFFI; G2, good GFFI; and G3, very good GFFI. Values are mean(SE).

^a^ p<0.05 versus G1.

^b^ p<0.05 versus G2.

As expected, the VO_2_max was different among groups. As shown in table 2, the values of VO_2_max in G3 were higher than those found in G1.

**Figure 3:**
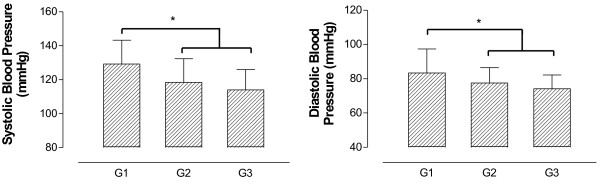
**Systolic and diastolic blood pressure in adults and elderly according to general functional fitness index results (G1 – regular GFFI; G2 – good GFFI; G3 – very good GFFI).** *p < 0.05.

**Figure 4:**
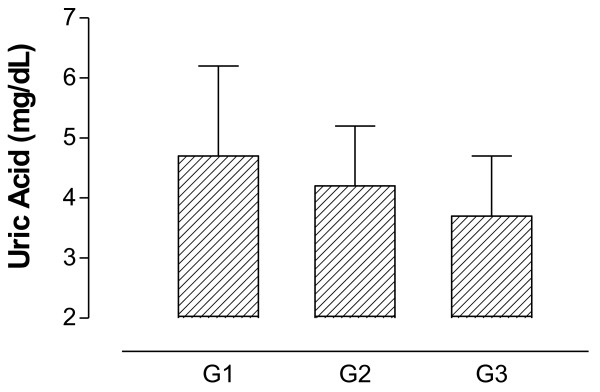
**Uric acid levels in adults and elderly according to general functional fitness index results (G1 – regular GFFI; G2 – good GFFI; G3 – very good GFFI).** *p < 0.05.

For lipid profile, CHOL and TG values were significantly lower between G2 and G3 compared to G1 and no difference was found in LDL-c and HDL-c among groups Figures 3 and 4 show BP and UA results. As shown in Figure 3, G2 and G3 presented lower values of SBP and DBP compared to G1. However, UA results were not different among groups (Figure 4).

**Figure 5:**
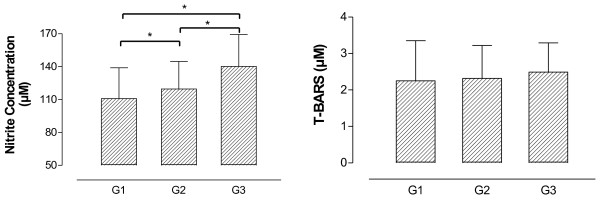
**Nitrite concentration and T-BARS results in adults and elderly according to general functional fitness index (G1 – regular GFFI; G2 – good GFFI; G3 – very good GFFI).** *p < 0.05.

Nitrite concentration and oxidative stress are other important variables that can explain the relationship between BP and UA Figure 5 illustrates that nitrite concentration was higher in G3 compared to G2, and G2 which was higher than G1. No difference was found in T-BARS analysis among groups.

Although the results indicate a relationship between the variables, the absence of differences in UA levels (Figure 4) could raise doubts regarding this relationship. Thus, Table 3 shows the results of BP when participants were divided according to the GFFI and UA.

**Table 3:** Blood pressure results of the participants divided according to GFFI and UA levels

	**Low levels of UA**	**High levels of UA**
Subjects classified as regular GFFI (G1)		
SBP (mmHg)	129.0 ± 17	129.6 ± 10
DBP (mmHg)	82.6 ± 16	84.4 ± 9.9
Subjects classified as good GFFI (G2)		
SBP (mmHg)	117.2 ± 15	125.7 ± 9.3
DBP (mmHg)	75.8 ±	87.2 ± 12.4
Subjects classified as very good GFFI (G3)		
SBP (mmHg)	111.8 ± 11.0	130.0 ± 8.8*
DBP (mmHg)	72.1 ± 7.3	89.0 ± 1.7*

*p<0.05 versus low level of UA.

It can be observed that only in G3 different levels of UA resulted in different values in BP. Therefore, this result suggests that very good levels of training status determine a reduction in BP values in the participants with low levels of UA.

## Discussion

The present study was designed to investigate the potential effect of a good level of training status on blood pressure and UA concentration and the main results found were that patients with higher training status and low level of uric acid have low values of blood pressure.

Uric Acid has an important influence on vascular control by increasing oxidative stress (O_2_^-.^ production) and NO-scavenging. This mechanism can affect the vasodilatation thereby increasing BP. Although it has been reported that physical exercise contributes to an adequate maintenance of BP, there is limited evidence demonstrating the effect of physical exercise on UA concentration.

Our results showed that subjects with elevated UA concentration demonstrated elevated BP. Also, we demonstrated that this result can be mediated by training status, since subjects with higher training status and lower levels of UA presented lower values of BP. The mechanisms that may explain the association between UA concentration and BP are still unknown. The anion superoxide (O_2_^·­^) produced during the UA formation acts as a NO-scavenging thus reducing the NO bioavailability. NO is a potent vasodilator and has an important function in BP control [27-30]. This is in agreement with Zharikov et al. [7], who have shown a widespread effect of inhibiting vascular endothelial NO production and accumulation of cyclic guanosine monophosphate (cGMP) without effect on endothelial nitric oxide synthase (eNOS) activity and expression. In accordance, Corry et al. (2008) have also demonstrated in cell cultures that the vascular smooth muscle cells from rat aorta decrease metabolic concentration of nitrite and nitrate and increase hydrogen peroxide (H_2_O_2_) and 8-isoprostane activity when UA (200 μmol) was added. The increased production of these compounds suggests that UA stimulates the production of reactive oxygen species (ROS) which are associated with impaired endothelium-dependent vasodilatation. Therefore, UA seems to be associated with decreased NO bioavailability and consequently increased BP [6]. In accordance, we have shown that nitric content was higher in the patients with high training status.

The results of this study showed no difference in T-BARS and UA concentration among groups. However, high level of training status resulted in better results of nitrite concentration and systolic and diastolic BP. Therefore, the mechanism responsible for better control of BP may be associated with better antioxidant capacity achieved by higher levels of training status and consequently, higher nitric oxide bioavailability.

It is worth noting that higher level of training status can only be achieved with regular physical training which is able to promote improvement in the antioxidant system. In fact, multicomponent exercise, including aerobic exercise, can enhance the mitochondrial respiration and it might contribute to a lower ADP/ATP ratio in skeletal muscle [31]. When ATP is used during exercise, AMP is degraded to IMP, hypoxanthine, xanthine and finally to UA. During exercise there is an increase of xanthine oxidase that contributes to the oxidative stress. However, this effect can be reduced by training status [32].

Waring et al. (2003) divided the participants with normal BP into two groups. The first group performed moderate-intensity exercise and the values of BP 40 min post-exercise were 147(6)/101(5) mmHg (SBP/DBP). The second group received oral administration of UA (0.5 g) before acute exercise and the values of BP 40 min post-exercise were 153(6)/103(6) mmHg (SBP/DBP). This study showed that an increased UA concentration might contribute to an increased BP after exercise. However, moderate-intensity exercise up-regulated the xanthine oxidase activity suggesting that a physiological level of UA might contribute to the BP control, especially because of the improvements in the antioxidative capacity [33].

Although regular exercise has been considered the major stimulus to prevent high blood pressure, there is still no consensus of which is the best combination among type, intensity and frequency to achieve such benefits. Thus, our study leads us to the discussion of how training status, regardless of how physical exercise was previously performed, exerts an important role on this mechanism associated with BP control.

The results presented in Table 2 showed that a higher level of functional fitness (G3) had a marked effect on CHOL and TG, when compared to a lower level of functional fitness (G1). Although LDL-c and HDL-c did not present significant difference among groups, lower results in LDL-c and higher results in HDL-c can be observed when training status is increased, suggesting that high GFFI may contribute to decrease the risk factor for cardiovascular disease. The lack of differences in LDL-c and HDL-c among groups can also be explained by the reference range established by the AHA [16]. It is important to note that the participants evaluated in this study did not present cardiovascular disease, and the values of lipid profile were within a range considered normal or borderline. Therefore, marked differences were not expected for these variables. However, it is important to assume that these effects would be very important for people with cardiovascular disease. Marques et al. [12] showed better results with multicomponent evaluation in TG and HDL-c when compared to one component (endurance). Kemmler et al. [10] also showed that a multipurpose exercise program significantly affected most parameters such as CHO, TG and HDL-c of the metabolic syndrome in elderly women. In addition, numerous physiological mechanisms may explain this alteration such as increased skeletal muscle lipoprotein lipase activity and increased capillary density [12,34].

## Conclusions

The present study found a relationship among SBP, DBP, UA, lipid profile and GFFI. Taking together the findings that subjects with elevated values of UA and lower values of nitrite had higher values of BP, this study suggests that the relationship between blood pressure and the oxidative stress produced by acid uric may be mediated by training status.

## Abbreviations

AAHPERD: American Alliance for Health Physical Education, Recreation and Dance; ACSM: American College of Sports Medicine; ADP: adenosine diphosphate; AHA: American Heart Association; AMP: adenosine monophosphate; ATP: adenosine triphosphate; BMI: body mass index; BP: blood pressure; CHOL: total-cholesterol; DBP: diastolic blood pressure; GFFI: eneral fitness functional index; HDL-c: HDL-cholesterol; LDL-c: LDL-cholesterol; NO: nitric oxide; NO2: nitrite; O2-: anion superoxide; ONOO-: peroxinitrite; SBP: systolic blood pressure; T-BARS: thiobarbituric acid reactive substances; TG: triglycerides; UA: uric acid;

$$\dot{V}O_{2}\max$$:

maximal oxygen uptake.

## Competing interest

“The authors declare that they have no competing interest”.

## Authors’ contributions

AAT, AMJ, JJM and JTCS have made substantial contributions to acquisition, analysis and interpretation of data. All of them have been involved in drafting the manuscript. JETS, SLA and ASZ have made substantial contributions to analysis and interpretation of data and have been involved in drafting the manuscript and revising it critically for important intellectual content. All authors read and approved the final manuscript.

## Limitations of the study

The authors understand that ambulatory BP measurements are more accurate than office BP measurements. However, we did not have this technique available at the time the data were collected; (b) although the authors are aware of the differences between genders, men and women were not analyzed separated in our study because no differences in all variables were found between male and female. We recognize the low number of men participating in the study; (c) the antihypertensive drugs were not interrupted during the study, however, participants were asked not to take the medicine before the blood pressure measurements; (d) some blood analysis such insulin levels were not performed; (e) no specific control of diet was performed, however, participants were requested to have a light meal before the test.

## Pre-publication history

The pre-publication history for this paper can be accessed here:

http://www.biomedcentral.com/1471-2261/13/44/prepub
